# Improving biomass and starch accumulation of bioenergy crop duckweed *(Landoltia punctata)* by abscisic acid application

**DOI:** 10.1038/s41598-018-27944-7

**Published:** 2018-06-22

**Authors:** Yang Liu, Xiaoyi Chen, Xinhui Wang, Yang Fang, Mengjun Huang, Ling Guo, Yin Zhang, Hai Zhao

**Affiliations:** 1 School of Pharmacy and Biological Engineering, Chengdu University, Chengdu, Sichuan 610106 China; 2 Key Laboratory of Environmental and Applied Microbiology, Chengdu Institute of Biology, Chinese Academy of Sciences, Chengdu, Sichuan 610041 China; 3 Environmental Microbiology Key Laboratory of Sichuan Province, Chengdu, Sichuan 610041 China; 4 Chongqing Key Laboratory of Environmental Materials & Remediation Technologies, Chongqing University of Arts and Sciences, 402160 Chongqing, China; 5 Meat Processing Key Laboratory of Sichuan Province, Chengdu, Sichuan 610106 China

## Abstract

Duckweed is a valuable feedstock for bioethanol production due to its high biomass and starch accumulation. In our preliminary experiment, we found that abscisic acid (ABA) could simultaneously increase starch and biomass accumulation of duckweed, but the mechanisms are still unclear. The results showed that the biomass production of duckweed reached up to 59.70 and 63.93 g m^−2^ in 6 days, respectively, with an increase of 7% (*P* < 0.05) compared to the control. The starch percentage increased from 2.29% up to 46.18% after 14 days of treatment, with a total of starch level 2.6-fold higher than that of the control. Moreover, the level of endogenous ABA, zeatin-riboside (ZR) and indole-3-acetic acid (IAA) increased, while gibberellins (GAs) decreased. Notably, ABA content in treated samples reached 336.5 mg/kg (fresh weight), which was 7.5-fold greater than that of the control. Importantly, the enzyme activities involved in starch biosynthesis increased while those catalyzing starch degradation decreased after ABA application. Taken together, these results indicated that ABA can promote biomass and starch accumulation by regulating endogenous hormone levels and the activity of starch metabolism related key enzymes. These results will provide an operable method for high starch accumulation in duckweed for biofuels production.

## Introduction

With an ever-increasing demands for energy, energy shortages have received increasing attention worldwide. Moreover, some nations still rely on the consumption of traditional resources, such as coal and crude oil, which may cause air pollution and other environmental problems^1^. Renewable and clean bioenergy will be an alternative resource to solve the energy crisis and environmental pollution. Bio-ethanol is a gasoline alternative that can reduce greenhouse gas (GHG) emissions when it is blended as an additive^2^. Therefore, it is urgent to explore novel and sustainable feedstocks (starch sources) for bio-ethanol production.

Duckweed, as the smallest flowering plants in the world, grows faster than most other higher plants^3^. Under ideal conditions, duckweed can double its biomass in 16–48 hours. The growth rate of duckweed can reach 12.4 g/m^2^/day dry weight, and its yield has been documented to reach 55 tons/hectare/year dry weight^4^. Duckweed can absorb nutrients such as nitrogen and phosphorus from wastewater, assimilating CO_2_ through photosynthesis and converting it into high-quality raw materials. Moreover, duckweed can accumulate starch up to 64.9% dry weight^5^. High starch accumulation but low lignin content has allowed successful conversion to bioethanol in recent years^6^. Therefore, duckweed is a promising new non-grain feedstock with starch that is expected to partially solve the problem that the fuel ethanol industry faces due to the lack of starchy materials.

The rapid accumulation of high-quality and high-quantity duckweed is considered the key step in its conversion to bio-ethanol. Previous studies have reported that many factors can affect duckweed biomass and starch accumulation, such as nutritional conditions (starvation stress)^7^, CO_2_ concentration^8^, plant hormones and light intensity^9–11^. High starch content can be realized under nutrient starvation stress, but cannot be used to treat sewage. It has been reported that elevated CO_2_ can also promote starch accumulation in duckweed. However, it is difficult to achieve large-scale outdoor production^8^. In addition, plant-growth regulators (PRGs) play an important role in plant growth and yield. In particular, ABA is a universal phytohormone involved in various physiological processes of plants, including seed development, improved growth and abiotic stress tolerance, and increasing carbohydrate accumulation in higher plants^12^. For example, the dry biomass yield was increased up to 2.1-fold, and saturated fatty acid content was promoted 11.17% compared with the control in *Scenedesmus quadricauda* after ABA supplement^13^. ABA seems to play an essential role in starch accumulation, but few studies have reported the application of ABA in duckweed. Wang^14^ has reported using ABA (added to cultures) to induce turion formation in *Spirodela polyrhiza*. However, turion is a special structure of *Spirodela polyrhiza* with high starch content, found at the bottom of water bodies, and would be difficult to harvest in future applications. Importantly, through a large number of pre-experiments, high starch accumulation of *Landoltia punctata* was obtained after spraying ABA on the surface of fronds.

This study shows for the first time that foliar spraying of ABA on the surface of *Landoltia punctate* increases starch accumulation in fronds. The objectives of this research were to determine the effect of ABA on the growth and the carbohydrate metabolism of duckweed and the underlying mechanism. The biomass of duckweed, starch accumulation, endogenous hormone content and key enzymes of carbohydrate metabolism were discussed. This work provides biochemical and physiological insights into *L. punctata* high-starch accumulation after ABA treatment. It also shows the dual benefits of duckweed: clean-up of sewage and production of potentially high-quality feedstock for biofuels.

## Results

### Effect of ABA on biomass accumulation of *L. punctata*

**Figure 1:**
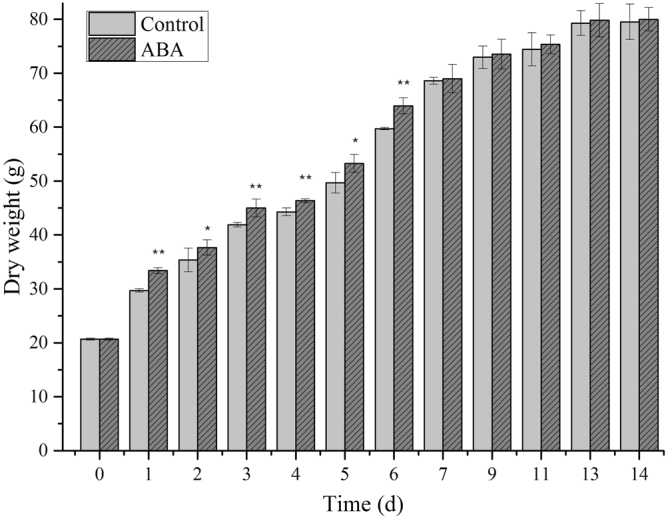
Dry weight accumulation of *L. punctata*. Each data point represents the mean of triplicate values; error bars indicate standard deviation. *And **indicate significant differences at the *P* = 0.05 and *P* = 0.01 probability level, respectively.

Application of ABA significantly affected biomass accumulation of *L. punctata*. The results showed that ABA can promote biomass yield compared with the control samples (Fig. 1). The dry weight of duckweed was 29.69 and 33.39 g m^−2^, with an increase of 12% in ABA treatment samples compared with controls on the 1st day (*P* < 0.05). By the 6th day, the biomass production of duckweed reached up to 59.70 and 63.93 g m^−2^, respectively, with an increase of 7% (*P* < 0.05) compared to that of the control (*P* < 0.05). At 14th day, the dry weight has reached a steady-state condition, with the dry weight of 79.51 and 79.98 g m^−2^, respectively.

### Impact of ABA on starch accumulation of *L. punctata*

**Figure 2:**
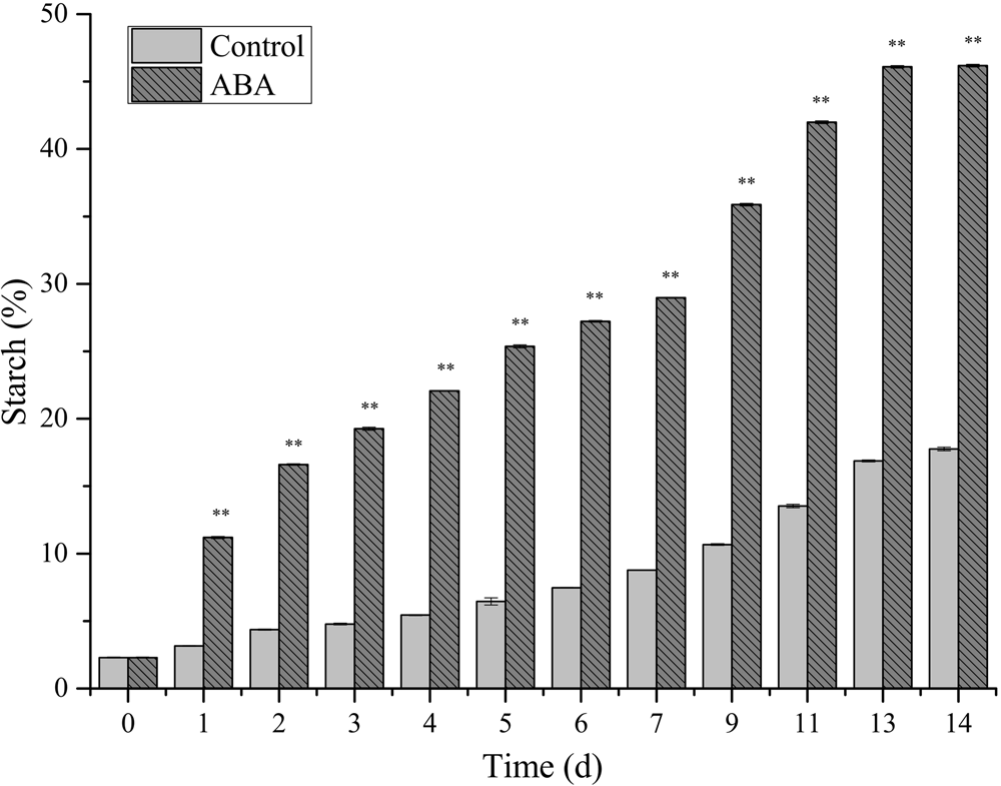
Starch percentage of ABA-treated *L. punctata*. Fronds were collected at different time points and used for starch percentage analysis. The starch percentage was calculated based on dry weight. Each data point represents the mean of triplicate values; error bars indicate standard deviation.

Besides biomass production, starch accumulation is another key consideration for duckweed application in biofuel production. As shown in Fig. 2, the results demonstrated that the starch content was 2.29% (DW) at 0 day, but reached 11.20% on the 1st day after ABA treatment. This content was 3.6-fold higher than the control sample, with a content of 3.12% (*P* < 0.05). The starch content reached 19.26% (DW) by the 3rd day and finally reached 46.18% (DW) after ABA treatment on the 14th day. However, in the control samples, the starch content remained steady at 4.78% by the 3rd day and 17.76% on the 14th day. On the last day, the content of treatment samples was 2.6-fold higher than that of the control (*P* < 0.05).

### Effect of ABA on *L. punctata* chlorophyll content and photosynthesis

**Figure 3:**
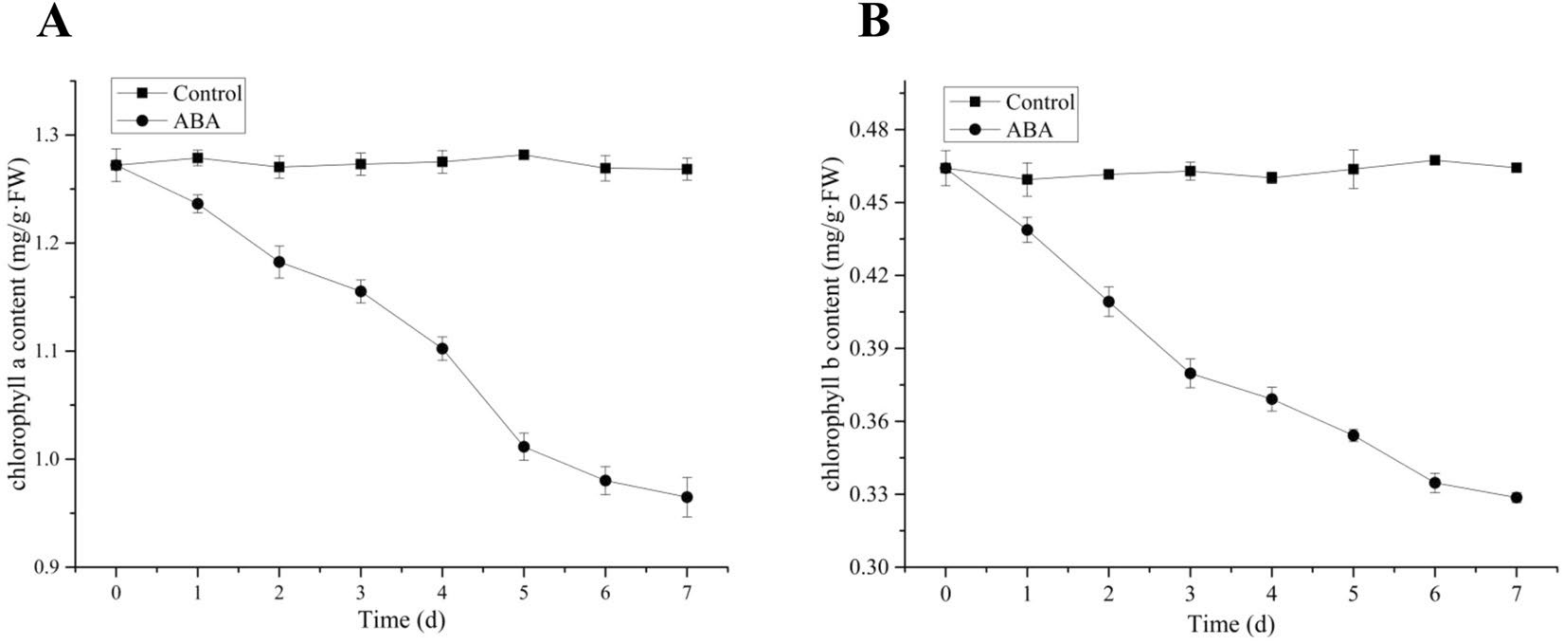
Chlorophyll a and chlorophyll b content of *L. punctata*. (**A**) Chlorophyll a content. (**B**) Chlorophyll b content. Values are given as the mean ± SD of three experiments for each group.

The effects of ABA application on photosynthetic pigments were also investigated in this study. As shown in the Fig. 3, chlorophyll a content decreased from an initial value of 1.27 to 0.96 mg/g (FW) following treatment, but there was little change in the control sample, which was 1.27 mg/g (FW) at day 0, and maintained at 1.26 mg/g (FW) at day 7. Similarly, chlorophyll b content decreased from an initial value of 0.46 to 0.33 mg/g (FW) following treatment. There was no change in the control samples.

**Figure 4:**
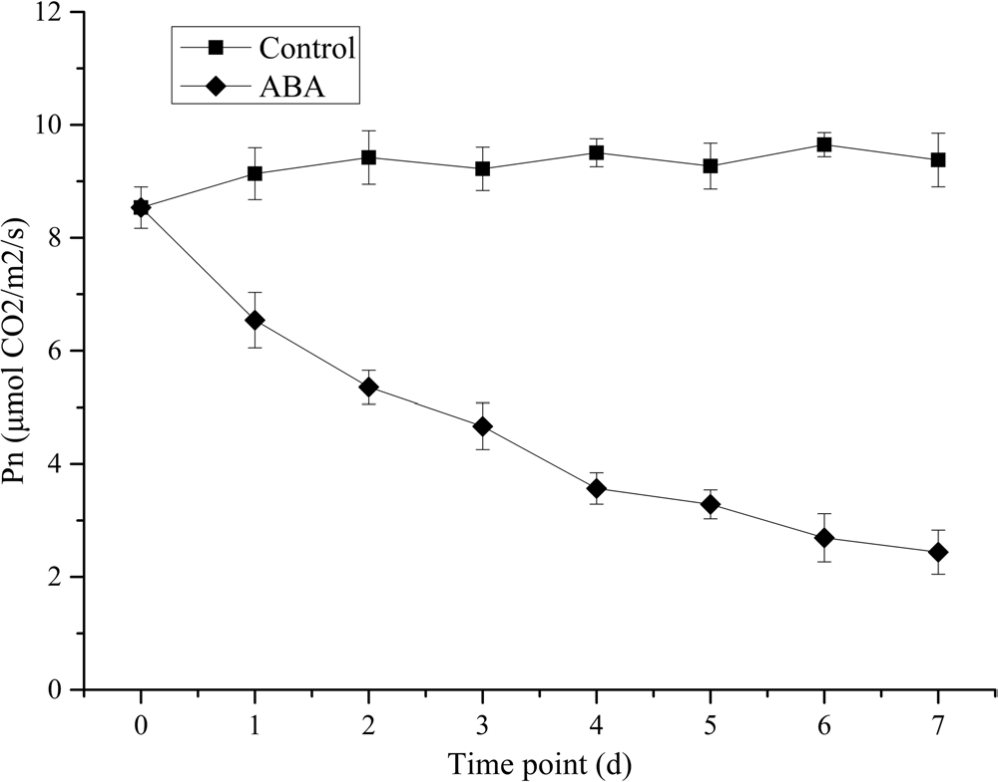
Effects of ABA treatment on net photosynthesis rate of duckweed. All data are presented as the mean of triplicate measurements ± standard deviation.

To investigate the effect of ABA on photosynthesis, net photosynthesis rates were also measured, as shown in Fig. 4. The results showed that the net photosynthesis rate slightly increased from 8.54 to 9.38 μmol CO_2_/m^2^/s and decreased to 2.44 CO_2_/m^2^/s in the control and the ABA treatment samples, respectively. During this period, ABA treatment obviously decreased the net photosynthesis rate of duckweed by 4.5-fold when compared with the control.

### Alteration of endogenous ABA, GAs, ZR and IAA levels in *L. punctata*

In this study, to analyze the response of endogenous hormones after ABA treatment, the levels of endogenous phytohormones, including ABA, GA1 + 3, ZR and IAA, were measured.

**Figure 5:**
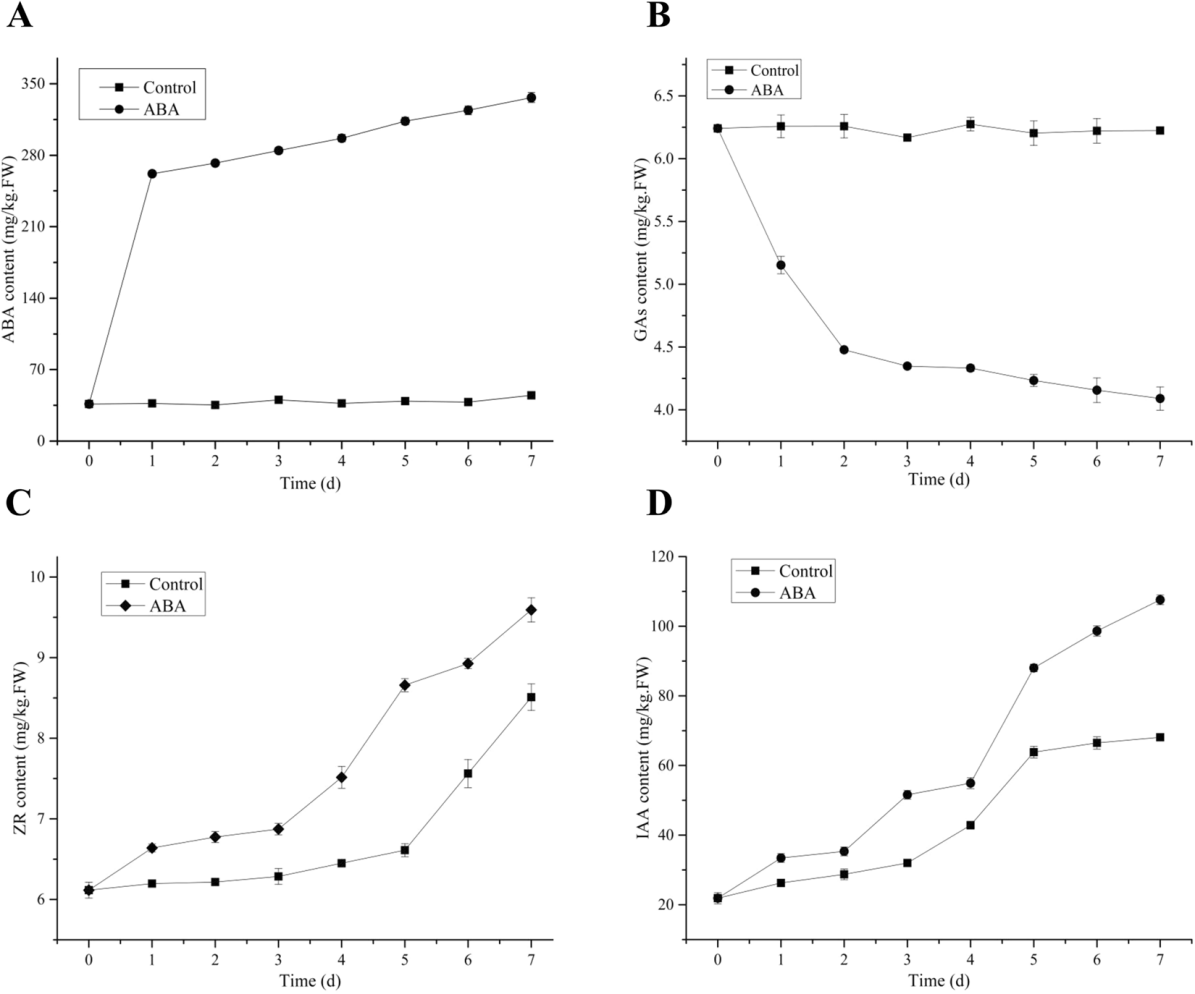
Effect of ABA treatment on endogenous ABA, GAs, ZR and IAA, levels in duckweed. (**A**) ABA content. (**B**) GAs content. (**C**) ZR content. (**D**) IAA content. FW, fresh weight. Values are given as the mean ± SD of three experiments for each group.

Endogenous ABA content rapidly rose from 36.3 to 262.0 mg/kg (FW) after ABA application. On the other hand, ABA levels were 37.0 mg/kg (FW) in control samples during the 1st day (Fig. 5A). By the 7th day, ABA content reached 336.5 mg/kg (FW) after ABA application, while there was little change in the control samples. Thus, endogenous ABA levels were 7.5-fold higher after ABA treatment than in the control samples. However, GA content decreased from 6.3 to 4.1 mg/kg (FW) following treatment, compared with 6.2 mg/kg (FW) in the control sample after 7 days of growth (Fig. 5B).

ZR (zeatin-riboside) content increased from 6.1 to 8.5 and 9.6 mg/kg (FW) in the control and ABA treatment samples, respectively (Fig. 5C). The level of ZR was much higher in the treatment sample than that of the control. As described in Fig. 5D, IAA content increased from 21.9 to 107.6 mg/kg (FW) after ABA application, which was 1.6-fold more than the control, with a content of 68.1 mg/kg (FW).

### Effect of ABA application on activities of enzymes involved in starch metabolism

**Figure 6:**
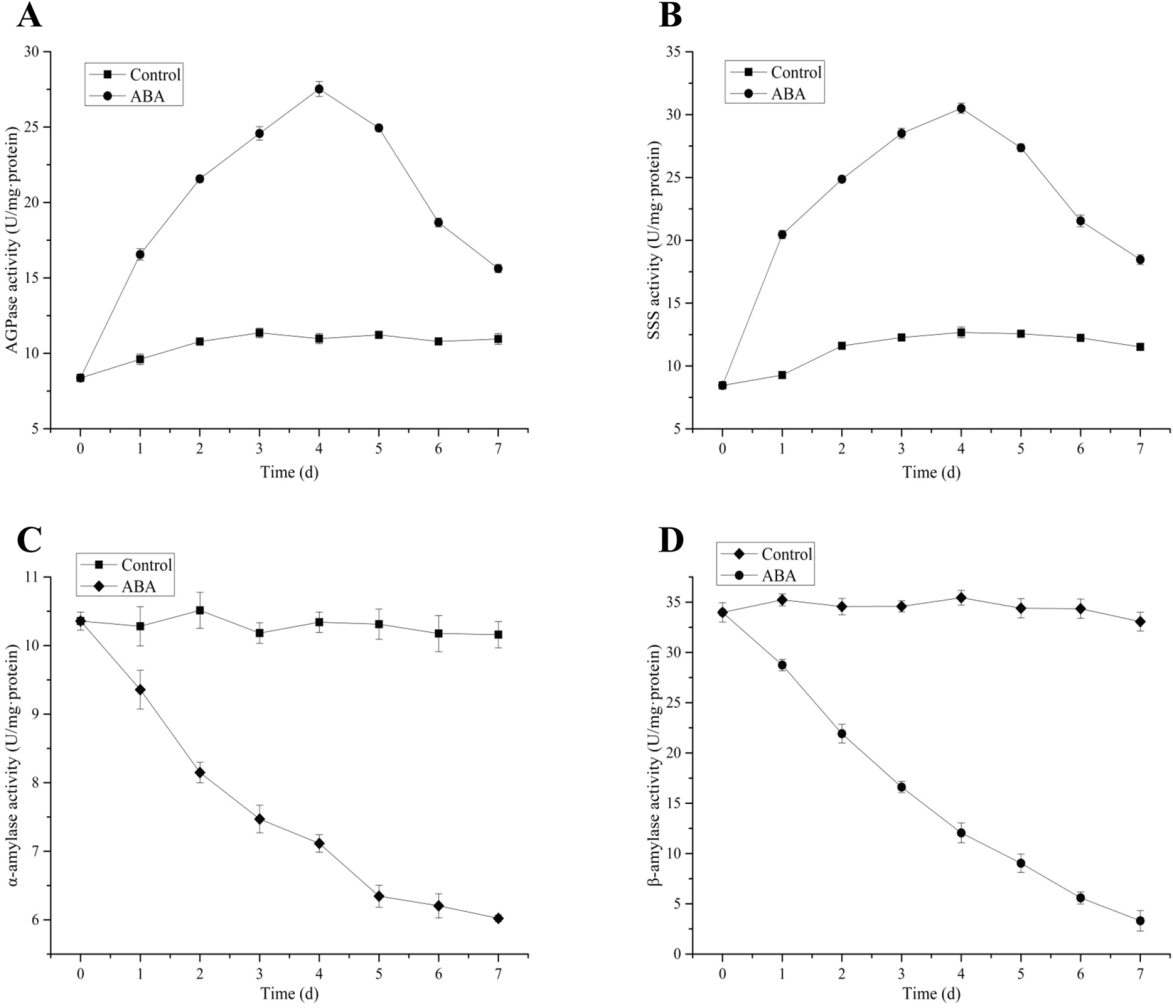
AGPase, SSS, α-amylase, and β-amylase activity. (**A**) AGPase activity; (**B**) SSS activity (**C**) α-amylase activity; (**D**) β-amylase activity. All data are presented as the mean of triplicate measurements ± standard deviation.

Several enzymes participate in the process of starch accumulation, and the activity of these enzymes seriously affects the accumulation of starch in plant. The key enzymes involved in starch biosynthesis (ADP-glucose pyrophosphorylase (AGPase) and soluble starch synthase (SSS)) and key enzymes (alpha-amylase (α-amylase) and beta-amylase (β-amylase)) related to starch catabolism were assayed in this study (Fig. 6).

The activity of AGPase increased from 8.37 to 10.98 U/mgprot (units per milligram protein) in the first 4 days in the control sample. It immediately rose from 8.37 to 27.53 U/mgprot after ABA application, which is a 2.5-fold increase compared with control samples (Fig. 6A). The activity of AGPase decreased slightly to 15.62 and 10.95 U/mgprot in ABA the treatment and control samples, respectively. The rising trend of SSS activity was similar to that of AGPase. SSS activity increased from the initial 8.46 to 30.50 U/mgprot in the first 4 days and then decreased to 18.47 U/mgprot by day 7 after ABA application. Meanwhile, SSS activity did not change much in the control samples (Fig. 6B).

Figure 6C,D showed the variation of α-amylase and β-amylase activities in different treatments. The activity of α-amylase gradually decreased after ABA application, while there was little change in the control samples. The activity of α-amylase decreased from 10.36 to 6.02 U/mgprot by the 7th day in the treatment samples. The activity of α-amylase reached 10.16 U/mgprot by the 7th day, which is 1.7-fold higher than that of the treatment samples (Fig. 6C). Additionally, the activities of β-amylase remarkably decreased from 33.98 to 3.31 U/mgprot by the 7th day, the final activity was 1/10 of the original activity. The activity of β-amylase maintained a stable level from 33.98 to 33.07 U/mgprot by day 7, which is 10-fold that of the ABA treatment samples (Fig. 6D).

## Discussion

Biomass production and starch accumulation are two key consideration in duckweed biofuel production. High-starch duckweed is a valuable feedstock for biofuel production. In this study, after culturing for 14 days, the starch content reached 46.18% in the treated samples and 17.76% in the control samples from an initial content of 2.30% (Fig. 2). As a result, combined with the biomass production the total starch content in the treated samples was 2.6 times higher than that in the control samples. The results are consistent with those obtained from other studies. For example, reports have shown that ABA can promote starch biosynthesis in rice culture cells^15^. In this study, both the biomass yield and starch accumulation were increased by ABA application compared to the control. Therefore, ABA application may be regarded as an effective method to increase starch and biomass accumulation for industrial duckweed cultivation.

Chlorophyll(Chl) is the most important photosynthetic pigment in the chloroplast of plants, which plays an essential role in absorbing light and transferring light energy in the antenna systems^16^. In this study, the results indicated that the chlorophyll content of duckweed decreased, triggered by exogenous ABA, and similar results were observed in other reports. ABA plays a positive role in the process of leaf senescence, and exogenously applied ABA can accelerate Chl degradation in Arabidopsis^17^. Moreover, it has long been known that exogenously applied ABA can induce the expression of senescence-associated mRNAs and can reduce chlorophyll content in detached leaves^18^.

Exogenous ABA application also decreased the net photosynthetic rate (*Pn*) in this study. These results are in agreement with those obtained by Li *et al*.^19^, who reported that the net photosynthesis rates decreased from 13.8 to 2.2 μmol CO^2^/m^2^/s after ABA treatment compared to the control of 13.5 μmol CO^2^/m^2^/s. In this study, the change of the net photosynthetic rate is consistent with the decrease of chlorophyll content.

Exogenous ABA can promote starch accumulation and affect the levels of endogenous phytohormones in plants. In this study, the level of endogenous ABA, ZR and IAA increased, while GAs decreased by ABA treatment (Fig. 5). The similar observations were also reported in other studies. Reports have shown that the expression of genes encoding starch degradation enzymes such as a-amylases and proteases were induced by GA but suppressed by ABA^20^. ABA promoted starch accumulation in rice cell cultures, and the gene expression of large subunits of ADPase was also up-regulated after ABA treatment^15^. Moreover, Wang^21^ investigated the physiological responses of kiwifruit plants to exogenous ABA under drought conditions and showed that ABA induced higher endogenous ABA content than the control after ABA treatment. Importantly, in this study, the increase in ABA and decrease in GAs also supported starch accumulation in duckweed. Therefore, ABA treatment might enhance starch accumulation by blocking synthesis of GAs and enhancing ABA biosynthesis in duckweed.

Cytokinins are a group of phytohormones that promote cell division and cell differentiation in plant roots and shoots but that also affect biomass and starch accumulation^22^. Hence, in this study, increased ZR promoted the biomass accumulation of duckweed by regulating cell division and differentiation. Moreover, a report has shown that ZR plays an important role in regulating starch accumulation patterns and consequently influences starch percentage^23^. The increase of ZR plays a role in promoting biomass yield and starch accumulation in duckweed. Indole-3-acetic acid (IAA) is considered to be the primary auxin in plants and stimulates growth through cell elongation and plant growth. In this study, the levels of IAA remarkably increased from 21.9 to 107.6 mg/kg (FW) after ABA application. The increase of ZR and IAA may be another important factor that influence duckweed biomass accumulation.

The alteration of the endogenous ABA, GAs, ZR and IAA co-regulates starch metabolism in *L. punctata*. The endogenous hormone content changed dramatically following ABA application (Fig. 6). In this study, the results showed that ABA treatment can promote AGPase and SSS activity. ADPglucose pyrophosphorylase (EC: 2.7.7.27) and soluble starch synthase (SSS, EC 2.4.1.21) are two of the most important key enzyme involved in starch synthesis. High activity of AGPase and SSS were beneficial to starch accumulation in duckweed. 4 genes encoding starch metabolism related enzymes were selected for qRT-PCR analysis (Supplementary Figure 1), and the primers of these genes were list in the Supplementary Table 1. The results showed that the expression patterns of these genes were consistent with the activities of enzyme activity data in this study. Moreover, some reports also showed that exogenous ABA application increased starch content by temporally regulating the expression levels of the starch synthesis enzyme genes, which are involved in starch biosynthesis, and their results supported the findings of this research^24,25^. For example, Wang *et al*.^24^ investigated the expression of ADP-glucose pyrophosphorylase (key enzyme involved in starch synthesis) during turion formation under ABA treatment in *Spirodela polyrhiza*. The results showed that the expression of *SpAPL2* and *SpAPL3* (subunits of ADP-glucose pyrophosphorylase) was dramatically increased by 2-and 10-fold in 1–3 days and leveling off after 5 days by ABA treatment, respectively. Thus, it is probably true in *Landoltia punctata* as well given that our data indicate that ABA treatment increased the activities of the enzymes involved in starch biosynthesis. Therefore, the increase of ABA content plays a pivotal role in starch biosynthesis by regulating the activities of key enzymes involved in starch biosynthesis. Therefore, the increase of ABA content plays a pivotal role in starch biosynthesis by regulating the activities of key enzymes involved in starch biosynthesis.

A-amylase and β-amylase involved in the process of starch degradation, which can dramatically affect starch content. A-amylase (EC 3.2.1.1) is a-1,4 endoglycolytic enzyme, responsible for the mobilization of stored starch reserves by initiating the degradation process. These genes encoding α-amylase have been shown to be differentially expressed at various developmental stages and environmental conditions through the action of plant hormones such as ABA. The expression levels of a-amylase is down-regulated by plant hormone, abscisic acid (ABA), during the process of starch metabolism^26,27^. Moreover, in this study, the activities of α-amylase and β-amylase were decreased dramatically by ABA treatment (Fig. 6). Reports showed that ABA can suppress the expression of genes encoding amylases, and β-amylase activity was negatively correlated with ABA concentration during grain development in barley^28,29^. Moreover, GAs can decrease starch synthesis and accumulation by inducing or activating α-amylase and other hydrolases^30^. Thus, the starch content in ABA treatment samples was much higher than that in the control sample, possibly due to an increase in the activity of starch biosynthesis enzymes and a drop in the activity of starch-degrading enzymes regulated by endogenous hormones in duckweed.

## Conclusion

In this study, high biomass yield and starch accumulation of *L. punctata* was achieved by ABA treatment. The increase of endogenous ZR and IAA enhanced the biomass yield of duckweed, and high levels of ABA promoted starch accumulation. Moreover, alterations of endogenous hormone levels also regulated the activity of enzymes involved in starch biosynthesis and catabolism in duckweed. These results evidenced the dual benefits of duckweed: clean-up of sewage and production of potentially high-quality feedstock for biofuels. It also provide an operable method for high starch accumulation in duckweed for biofuels production in the future.

## Materials and Methods

### Duckweed cultivation and ABA treatments

*L. punctata*, a widely distributed duckweed species with great potential to accumulate enormous amounts of starch, originally isolated from Sichuan Province, China, was cultivated in standard 1/6 Hoagland’s E + solution in illuminating incubator (Guangzhi Corp. China) for 3 to 5 days (16 h 130 μmol/m^2^/s 25 °C: 8 h 0 μmol/m^2^/s 15 °C). Then 9 g of fronds were transferred into 1,400 mL 1/6 Hoagland E + culture plastic containers (25*18*4.5 cm) for further cultivation over a period of 14 days treatment. 5 mL solution of 0.5 mM ABA (Sigma-Aldrich, St Louis, USA) was sprayed evenly on the surface of fronds. For the control group, there was no plant hormones application on the surface of the fronds. Three biological replicates were performed in the experiment, and the experiment lasted for 14 days.

### Determination of fresh and dry weights

To measure the fresh weight (FW), the fronds were measured with a balance using Bergmann’s method^31^. To determine the dry weight (DW), the samples were dried at 60 °C until the weight was constant.

### Determination of starch content

The starch content was determined according to previously published methods^32^. Dry duckweed powder (0.03 g) was hydrolyzed with 2.6 mL 1.2 M HCl in a boiling water bath for 2 h. Then adjusting the pH to 7.0 with NaOH, adding PbAc to precipitate protein and diluting the solution with deionized water to a final volume of 10 mL. Thereafter, the solution was filtered and treated with a C18 extraction column. Finally, the hydrolyzate was analyzed by HPLC (Thermo 2795, Thermo Corp.) with an Evaporative Lightscattering Detector (All-Tech ELSD2000, All-tech., Corp.). The starch content was determined using the total sugar content (starch content = glucose content × 0.909).

### Total photosynthetic pigment analysis

The chlorophyll content was estimated according to Aron’s method^33^. Weigh 0.5 g of duckweed sample in a 10 ml plug seal centrifuge tube, 10 ml of 80% acetone was added and kept it 72 h at room temperature in dark condition. After the incubation, the supernatant was collected and then optical density was measured at 645 and 663 nm for chlorophyll a and chlorophyll b respectively.The chlorophyll content was expressed as chlorophyll mass on a fresh weight (FW) basis (mg/g FW).

The net photosynthesis rate was measured by Li-6400xt (LI-COR, Inc., St. Lincoln, NE, USA) using the whole chamber (6400–17) and the RGB light source (6400–18).

### Endogenous level of plant hormones

Duckweed was rinsed with distilled water using a strainer for three times. After free water stopped dripping, duckweed was blotted dry with paper towels, and then measured with a balance. The extraction, purification, and determination of endogenous levels of ABA, GA1 + 3, ZR and IAA by an indirect ELISA technique were performed as described by Wang^34^ and Yang^35^ with appropriate improvements. 0.5 g of fresh duckweed samples were homogenized in 2 ml of 80% methanol (containing 40 mg l^−1^ butylated hydroxytoluene as an antioxidant). The extract was incubated at 4 °C for 48 h, and then centrifuged at 4000 rpm for 15 min at 4 °C. The supernatant was passed through C18 Sep-Pak cartridges (Waters Corp., Millford, MA, USA), and the phytohormone fraction was eluted with 10 ml of 100% (v/v) methanol and then 10 ml of ether. The eluate was dried down by pure N_2_ at 20 °C, and then stored at −20 °C until use for endogenous hormone assay.

### Carbohydrate metabolism enzyme activity assay

For the assays of enzymes, 1 g fresh weight duckweed was homogenized with a ceramic pestle in an ice-cold mortar in 5 ml of 50 mmol/L HEPES-NaOH (pH = 7.6), 5 mmol/L DL-Dithiothreitol, 8 mmol/L MgCl_2_, 2 mmol/L EDTA, 2%(w/v) polyvinylpyrrolidone-40, 12.5% (w/v) glycerol. The homogenate was centrifuged at 10,000 g for 5 min. The supernatant extract was used as crude enzyme solution stored at minus 20 °C. All the procedures were carried out at 0–4 °C. Then, the supernatant was used to measure the enzyme’s activity. The activities of ADP-glucose pyrophosphorylase (AGPase, EC 2.7.7.27), soluble starch synthase (SSS, EC 2.4.1.21), alpha-amylase (α-amy, EC 3.2.1.1) and beta-amylase (β-amy, EC 3.2.1.2) were analyzed using the method from a previous report^36–38^. Activity was expressed as units per milligram protein (U/mgprot). Accordingly, one unit of AGPase is defined as the amount of enzyme that causes the increase of 0.01 OD at 340 nm of the finally reacted solution per minute. The AGP units are then divided by the total protein amount. One unit of amylase is defined as the amount of enzyme in the presence of excess thermostable a-glucosidase required to release 1 μmol of *p*-nitrophenol from non-reducing-end blocked p-nitrophenyl maltoheptaoside (BPNPG7) per min under the defined assay conditions. One unit of SSS is defined as the amount of enzyme that causes the increase in absorbance at 340 nm of the finally reacted solution per minute.

### Calculations and statistics

All samples were investigated in triplicate. All the results are presented as means ± standard error in the Figures. Statistical significance (*p* < 0.05) was analyzed by one-way analysis of variance^39^ using SPSS software (Version 15.0, SPSS Inc., Chicago, IL, USA).

## Electronic supplementary material

Supplementary table and figure
