# Supplementary material for: Improving biomass and starch accumulation of bioenergy crop duckweed (Landoltia punctata) by abscisic acid application
Source: Sci Rep. 2018 Jun 22;8:9544. doi: 10.1038/s41598-018-27944-7 (PMC6015002; doi:10.1038/s41598-018-27944-7)
Supplement: Supplementary file 1 — Supplementary table and figure [file 41598_2018_27944_MOESM1_ESM.doc]

**Improving biomass and starch accumulation of bioenergy crop duckweed *(Landoltia punctata)* by abscisic acid application**

Yang Liu^1^, Xiaoyi Chen^1^, Xinhui Wang^1^, Yang Fang^2,3^,, Mengjun Huang^4^, Ling Guo^2,3^, Yin Zhang ^1,5^, Hai Zhao^2,3^*

^1^ School of Pharmacy and Biological Engineering, Chengdu University, Sichuan 610106, China

^2^ Key Laboratory of Environmental and Applied Microbiology, Chengdu Institute of Biology, Chinese Academy of Sciences, Chengdu, Sichuan 610041, China

^3^ Environmental Microbiology Key Laboratory of Sichuan Province, Chengdu, Sichuan 610041, China

^4^ Chongqing Key Laboratory of Environmental Materials & Remediation Technologies, Chongqing University of Arts and Sciences, 402160, Chongqing

^5^ Meat Processing Key Laboratory of Sichuan Province, Sichuan 610106, China

*Corresponding author: Hai Zhao: zhaohaicib@126.com

E-mail address: Yang Liu: shuimu119@126.com

Xiaoyi Chen: xiaoyichenbio@126.com

Xinhui Wang: wangxinhui19820319@163.com

Yang Fang: fangyangcib@126.com

Mengjun Huang: huangmj806@gmail.com

Ling Guo:glby@163.com

Yin Zhang: zhangyincdu@126.com

Supplementary Table 1 The primers of genes related with starch metabolism

| Primer name | Primer F | Primer R |
| --- | --- | --- |
| *LeAPL1* | GCAGATGTAGTTCGCCAGTTT | GTTGTTTCCCTCCCATAATAGG |
| *LeAPL2* | GGGAGGACATAGGGACGATAAA | CAGAAAGCGAGGAGAGGTGAAG |
| *LeAPL3* | TCGTCCGTCTCCTTCCCTTC | GCGGAACTGGGCTGAAACTC |
| *LeAMY* | TCCCGCTGTAAATAAGGTGGC | GGGGGCATTCTTGGCAT |
| β-actin | GAATGGGACAGAAGGATGCG | TTCGGTGAGAAGAATAGGATGCT |

The large subunits of AGPase gene (*LeAPL1, LeAPL2, and LeAPL3*), a-amylase gene (*LeAMY*). The primers sequences for *LeAPL1, LeAPL2, LeAPL3* and *LeAMY* according to our previous experiment ^7^.


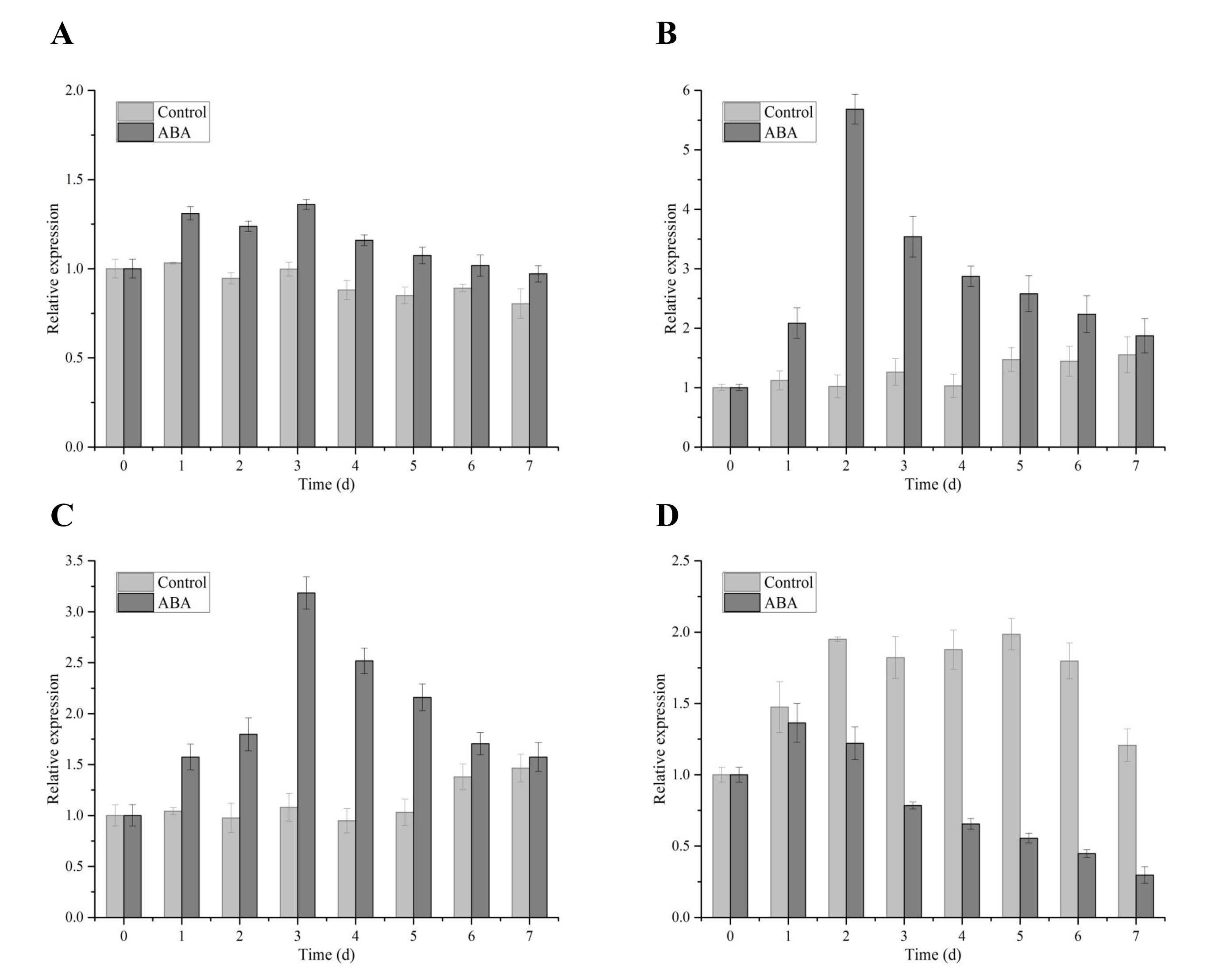


Supplementary figure 1. Expression of the three large subunits of AGPase gene-*LeAPL1* (A), *LeAPL2* (B), *LeAPL3* (C), and a-amylase gene-*LeAMY* (D) over time in *L. punctata* during ABA treatment and in controls. Relative expression was quantified by qRT-PCR and normalized to β-actin. Each bar represents three repetitions sample. Error bars representing standard errors are shown in each case.
